# In vivo optoacoustic imaging of endothelin receptor expression and treatment response in the hypoxic tumor microenvironment

**DOI:** 10.1007/s00259-025-07494-7

**Published:** 2025-08-13

**Authors:** Carsten Höltke, Moushami Mallik, Miriam Stölting, Emily Hoffmann, Christiane Geyer, Raghu Erapaneedi, Friedemann Kiefer, Anne Helfen

**Affiliations:** 1 Clinic for Radiology, University of Muenster and University Hospital Muenster, Muenster, Germany; 2 European Institute for Molecular Imaging (EIMI), Multiscale Imaging Centre (MIC), University of Muenster, Muenster, Germany

**Keywords:** Tumor hypoxia, Endothelin system, Tumor microenvironment, Optoacoustic tomography, In vivo imaging

## Abstract

**Purpose:**

A hypoxic tumor microenvironment promotes cancer progression, with endothelin-A receptor (ET_A_R) signaling playing a key role in tumor neoangiogenesis and macrophage infiltration. We hypothesize that multispectral optoacoustic tomography (MSOT) using an ET_A_R-specific probe could provide improved insights into the hypoxic characteristics of the tumor microenvironment (TME), either alone or in combination with endogenous markers, and that alterations in ET_A_R expression may correlate with increased tumor oxygenation serving as an early indicator of response to anti-angiogenic or immune-modulating therapy.

**Methods:**

A fluorescent ET_A_R probe was applied for in vivo MSOT evaluation of ET_A_R expression in hypoxic murine breast cancer. Optoacoustic signal intensity (SI) of deoxygenated and oxygenated hemoglobin served as additive intrinsic readouts. Furthermore, therapeutic interventions utilizing *Bevacizumab*, *Clodronate* and *Sorafenib* were evaluated with regard to effects on ET_A_R expression and hemoglobin oxygen saturation. Imaging results were validated ex vivo via immunohistochemistry.

**Results:**

Exposure of 4T1 murine breast cancer cells to hypoxic conditions led to upregulation of ET_A_R in vitro. In vivo, tumor growth correlated with increased ET_A_R probe signal intensity in 4T1 tumors. All therapeutic interventions significantly reduced ET_A_R SI following treatment. Anti-angiogenic therapies also increased tumor oxygen saturation, indicating therapy-induced re-oxygenation.

**Conclusion:**

ET_A_R expression in hypoxic tumor regions can be visualized non-invasively by MSOT using an exogenously administered targeted probe. Combining ET_A_R-targeted imaging with intrinsic hemoglobin readouts enables assessment of reoxygenation and immune cell modulation in response to therapy. Thus, ET_A_R has potential as an in vivo imaging biomarker for early therapy response in experimental breast cancer studies.

**Supplementary Information:**

The online version contains supplementary material available at 10.1007/s00259-025-07494-7.

## Introduction

A hypoxic tumor microenvironment (TME) is a fundamental contributor to tumor progression and malignancy, resulting from uncontrolled and dysregulated cellular growth and proliferation, leading to a discrepancy between oxygen availability and consumption [1–3]. Furthermore, a hypoxic TME is important during the establishment of a permissive and immunotolerant cancer phenotype, inducing pro-tumoral polarization of macrophages (tumor-associated macrophages– TAMs) [4, 5]. The cellular response to hypoxia is principally relayed through hypoxia-inducible factors (HIFs). HIFs are transcription factors activated by impaired oxygen supply, e.g. within the TME [3, 6]. In the human breast cancer cell line MDA-MB 231 more than 1,300 genes were found to be upregulated in a HIF-dependent manner [7]. Among these hypoxia-responsive genes are pro-angiogenic factors like vascular endothelial growth factor A (VEGF-A), immune checkpoint molecules like PD-L1 [8] and components of the endothelin (ET)-axis like the endothelin-converting enzyme-1, the endothelin-A receptor (ET_A_R) and the prevalent peptide endothelin-1 (ET-1) [9]. ET-1 is a small peptide mainly produced by vascular endothelial cells but also by tumor cells [10]. A large number of human cancers display an enhanced expression of ET-axis components, including ET-1 and the endothelin receptors ET_A_R and ET_B_R [11, 12]. ET-1, by signaling through ET_A_R, serves as both a growth and a survival factor for tumors, affecting cell proliferation, invasion and migration, promoting drug resistance, dissemination and angiogenesis as well as lymphangiogenesis and immune evasion [13]. ET-1 itself is able to stabilize HIFs by regulating prolyl hydroxylase activity, thereby preventing HIF degradation [9]. In murine tumor models, co-localization of ET-1 and hypoxic areas within primary tumors has been observed, especially in areas adjacent to necrotic lesions [14]. Overexpression of ET_A_R and its regulation by hypoxia in vitro has been described in e.g. breast cancer tissue [15] as well as in ovarian cancers [16].

Recently, multispectral optoacoustic tomography (MSOT) has been identified as a powerful biomedical imaging technology that allows non-invasive monitoring of (patho)physiological processes in vivo. It combines the high spatial resolution and deep tissue penetration of ultrasound imaging with the molecular sensitivity and specificity of optical (fluorescent) imaging. This makes MSOT superior to conventional fluorescence imaging, especially for visualizing deeper-lying tissues. Additionally, because it operates without radioisotopes, MSOT reduces regulatory and safety concerns associated with traditional nuclear imaging techniques [17]. By allowing spectral unmixing of optoacoustic signals, MSOT enables simultaneous detection and quantification of endogenous fluorophores such as hemoglobin or melanin, as well as exogenously administered targeted fluorescent probes. Collected data are reconstructed into a 3D volume image, depicting e.g. a subcutaneous tumor in preclinical cancer research [18]. Applications of this technique have already been translated to first clinical trials, where the assessment of blood oxygenation or tissue abnormalities yields an additional diagnostic value. This includes imaging of thyroid [19] and breast tumors [20, 21], detection of neuromuscular diseases [22] as well as diagnosis of vascular abnormalities such as venous and arteriovenous malformations [23]. The ability of MSOT to follow tumor growth and the treatment response in murine xenograft models by specifically discriminating and determining oxygenated (HbO_2_) and deoxygenated hemoglobin (Hb) within the hypoxic TME has recently been shown by Karlas et al. [24] and Quiros-Gonzalez et al. [25].

In this study, we aimed to determine whether non-invasive optoacoustic imaging in vivo with an exogenous ET_A_R-targeted fluorescent probe, used alongside endogenous markers, can provide enhanced insights into the hypoxic TME. We specifically assessed whether changes in ET_A_R expression as detected by MSOT correlate with variations in tumor oxygenation. Furthermore, we evaluated the potential of this probe as an imaging biomarker for the early assessment of treatment response to anti-angiogenic and immune-modulating therapies. Our findings could facilitate improved monitoring of the TME and support the development of personalized therapeutic strategies.

## Methods

### Probe

In addition to measuring the endogenous fluorophores hemoglobin and oxygenated hemoglobin, an exogenous small molecular fluorescent probe targeting ET_A_R was applied. This probe was labeled with IRDye800cw (LI-COR Biotechnology GmbH, Bad Homburg, Germany), which has an absorption maximum at 774 nm in phosphate-buffered saline (PBS) and was previously evaluated in a murine model of myocardial infarction [26].

### Tumor model

As a syngeneic tumor model, the murine breast cancer wildtype cell line 4T1 (CRL-2539, ATCC, Manassas, Virginia, USA) with high malignant potential (rapid local growth, lymphatic and hematogenous metastasis) was used; cells were cultivated as previously described [27]. Prior to injection, the cells were trypsinized (0.05% trypsin-EDTA, Gibco, Thermo Fisher Scientific, Waltham, MA, USA) and resuspended in medium.

### Animals

Mice (BALB/c) were purchased from Charles River Laboratories (Sulzfeld, Germany) and kept in groups of up to 5 animals in pathogen-free macrolon cages with filtered lids and provided with enrichment material, housed at constant temperature (22 ± 2 °C) and relative humidity (46 ± 4%) in a rodent flow cabinet. Animals had free access to water and food (normal chow diet, 1324 Best, Altromin, Lage, Germany). All animal experiments described in this study were approved by the responsible authorities (“Landesamt für Natur, Umwelt und Verbraucherschutz NRW”, Germany, Protocol No. 84-02.04.2017.A011). Tumor cells (0.5 × 10^6^) were orthotopically implanted via injection into the lower left mammary fat pad using extra fine 29G syringes. Tumor sizes were measured regularly using a digital caliper or ultrasound. To minimize tumor size effects, imaging experiments were initiated once tumors reached a maximum diameter of 4 mm.

For imaging, mice were shaved on the lower abdomen and randomly assigned to 6 experimental groups. Group 1 was used to determine the optimal probe concentration and imaging time points. Mice received intravenous injections of 2.0 nmol, 5.0 nmol and 10.0 nmol of the fluorescent probe via the tail vein and were imaged prior to, and 3 h, 6 h, 24 h and 48 h post injection (*n* = 5 per probe amount, imaged longitudinally). Based on these experiments, a dose of 5.0 nmol per animal with imaging at 24 h post injection was selected as optimal. Group 2 animals received 5.0 nmol of the probe and were imaged at the 24 h time point to record additional baseline data (total *n* = 12). Groups 3–6 were assigned to receive various anti-tumor therapies detailed in Fig. S1 (see supplementary information). Immediately following the initial imaging session, treatment regimens were started as follows: Group 3, *Clodronate*-loaded liposomes (Liposoma BV, Amsterdam, Netherlands) at 50 mg/kg body weight i.v. every two days and group 4, PBS-loaded liposomes (Liposoma BV) as a control. Group 5, *Sorafenib* (Selleck Chemicals, Houston, USA) 30 mg/kg body weight by daily oral gavage; and group 6, *Bevacizumab* (Avastin, Roche, Basel, Switzerland) at 5 mg/kg body weight intravenously every two days. Mice from all groups were imaged at days 4 and 8 after therapy initiation, with imaging performed 24 h after probe injection. Following the final imaging session, mice were sacrificed and tissues were harvested for further analysis as described below.

### Imaging

We used a commercial small animal imaging system (MSOT inVision 512-echo, iThera Medical GmbH, Munich, Germany), capable of simultaneous ultrasound and optoacoustic delineation of tissue. The system has been described in detail elsewhere [28]. Briefly, a tunable (680–1300 nm) optical parametric oscillator, pumped by a pulsed Nd: YAG laser, with 10 Hz repetition rate and up to 7 ns pulse duration is used for signal excitation. Light is delivered to the sample through a custom optical fiber assembly to obtain a uniform diffuse ring of illumination over the imaging plane. Coupling of the sample to the transducers is achieved using a water bath filled with deionized water. An array of transducers with a curvature radius of 4 cm covers an angle of 270° and allows tomographic reconstruction of the collected signals. Animals were placed in the provided animal holder (iThera Medical) and wrapped in a thin polyethylene membrane with the regions of interest covered in ultrasound gel (Aquasonic Clear, Parker Labs, Fairfield, NJ, USA). The holder was then assembled within the imaging system and immersed in deionized water maintained at 37 °C. During examination, mice were under inhalation anesthesia (isoflurane 1.2% in 1.0 l/min oxygen). Animal respiration was monitored during the imaging procedure using an integrated system camera. Serial images were acquired with 500 μm step width at excitation wavelengths 680, 700, 730, 760, 800, 850, 880, 910 and 940 nm, with an average of 10 frames per wavelength.

### Imaging data analysis

MSOT images were reconstructed and signal intensity data extracted using algorithms available in the ViewMSOT 4.0 software (iThera Medical); with fluence correction and spectral unmixing performed using the software’s embedded methods. Initially, various reconstruction and spectral unmixing methods were evaluated for accurate assessment of signal intensity. Only model-based reconstruction and guided ICA (independent component analysis) yielded reasonable values, while basic backprojection and linear regression algorithms failed to reliably detect hypoxia within lesions, as evidenced by inappropriately elevated sO_2_ values (Fig. S2b). Additionally, signal intensity values for IRDye as exogenous fluorophore ranged from 10^−5^ to 10^−4^, which were significantly below detection thresholds (data not shown). Anatomical ultrasound images were used for tumor localization and to ensure accurate spatial mapping. Regions of interest (ROIs) were manually delineated around tumors in the XY plane across consecutive cross sections to generate volumes of interest, that encompassed each lesion. For comparison, a random subset of animals was used to record contralateral muscle tissue data (*n* = 8). Spectral unmixing provided channel specific intensity measurements for oxygenated hemoglobin (HbO_2_), deoxygenated hemoglobin (Hb) and for IRDye800cw (IRDye) within these volumes. Total hemoglobin (HbT = Hb + HbO_2_) and tissue oxygenation saturation (sO_2_ = HbO_2_/HbT) were calculated using the ViweMSOT software. Data are displayed as mean ± SEM.

### Real time PCR and Western Blotting

Following treatment, normoxic and hypoxic 4T1 cells were analyzed by real time PCR and western blotting to assess gene and protein expression levels, respectively. Detailed protocols are provided in the supplementary information section.

### Immunohistochemistry

Tumor samples were preserved as cryoblocks and sectioned at 5 µm thickness using a Leica CM1950 cryostat (Leica, Wetzlar, Germany). Cryosections were air-dried and incubated in PBS at 37 °C for 10 min to remove OCT compound residues. Sections were then fixed in 4% PFA at RT for 20 min and permeabilized with 0.2% Triton X-100 in PBS for 25 min. Antigen retrieval was performed using Vector 3300 unmasking solution (Vector Laboratories Inc., Burlingame, CA, USA) according to the manufacturer’s instructions. Hematoxylin and eosin (H&E) staining was performed to examine tumor morphology. For immunofluorescence, sections were incubated with primary antibodies against carbonic anhydrase IX (CAIX, ABIN363423, antibodies online), endothelin-A receptor (ET_A_R, ab76259, Abcam, Cambridge, UK), PECAM-1 (ab124432, Abcam) and CD68 (ab125212, Abcam). Following incubation, sections were treated with a goat anti-rabbit AlexaFluor647-conjugated secondary antibody (#111-605-144; Jackson ImmunoResearch Europe Ltd. Ely, UK). Nuclei were counterstained with DAPI (4’,6-diamidin-2-phenylindol; #62248, Thermo Fisher Scientific). Appropriate negative controls were included in all staining procedures.

For additional analysis of ET_A_R and CAIX, tissue samples were fixed in 4% neutral-buffered formalin for 24 h and subsequently embedded in paraffin according to standard protocols. Paraffin Sect. (5 μm thickness) were cut using a Leica RM2235 microtome (Leica, Wetzlar, Germany). Immunohistochemistry for ET_A_R (ab76259, Abcam, Cambridge, UK) was performed using a Vectastain ABC kit (PK-6101, Vector Labs, CA, USA) and visualized with DAB peroxidase substrate (SK-4100, Vector Labs, CA, USA). Negative controls were prepared for each staining series.

Sections were imaged using a Nikon Eclipse 50i microscope and documented with NIS-Elements Br 3.22 software (Nikon Corporation, Tokyo, Japan). Tumor vascularization was determined by estimating the area coverage positive for PECAM-1 in tumor sections (see supplementary information for details).

### Statistical analysis

Data were analyzed using GraphPad Prism software (Version 7.05, GraphPad Software Inc.). All data sets were assessed for normal distribution using either D’Agostino & Pearson or Shapiro-Wilk normality tests, and not all passed. Therefore, where appropriate, respective corrections such as Welch’s t-test were applied. One-way ANOVA and unpaired t-test analyses were used to determine significant differences (*p* < 0.05). Post hoc tests were applied where appropriate to further evaluate pairwise comparisons following ANOVA. Data are displayed as box-and-whisker plots with mean signal intensity (SI) displayed on the Y-axis in arbitrary units (au). The baseline data presented is a summation of data from baseline animals (*n* = 12) and initial data of the therapy groups 3–6 (*n* = 48).

## Results

### Endothelin receptor expression is elevated in 4T1 breast cancer cells under hypoxia

**Fig. 1:**
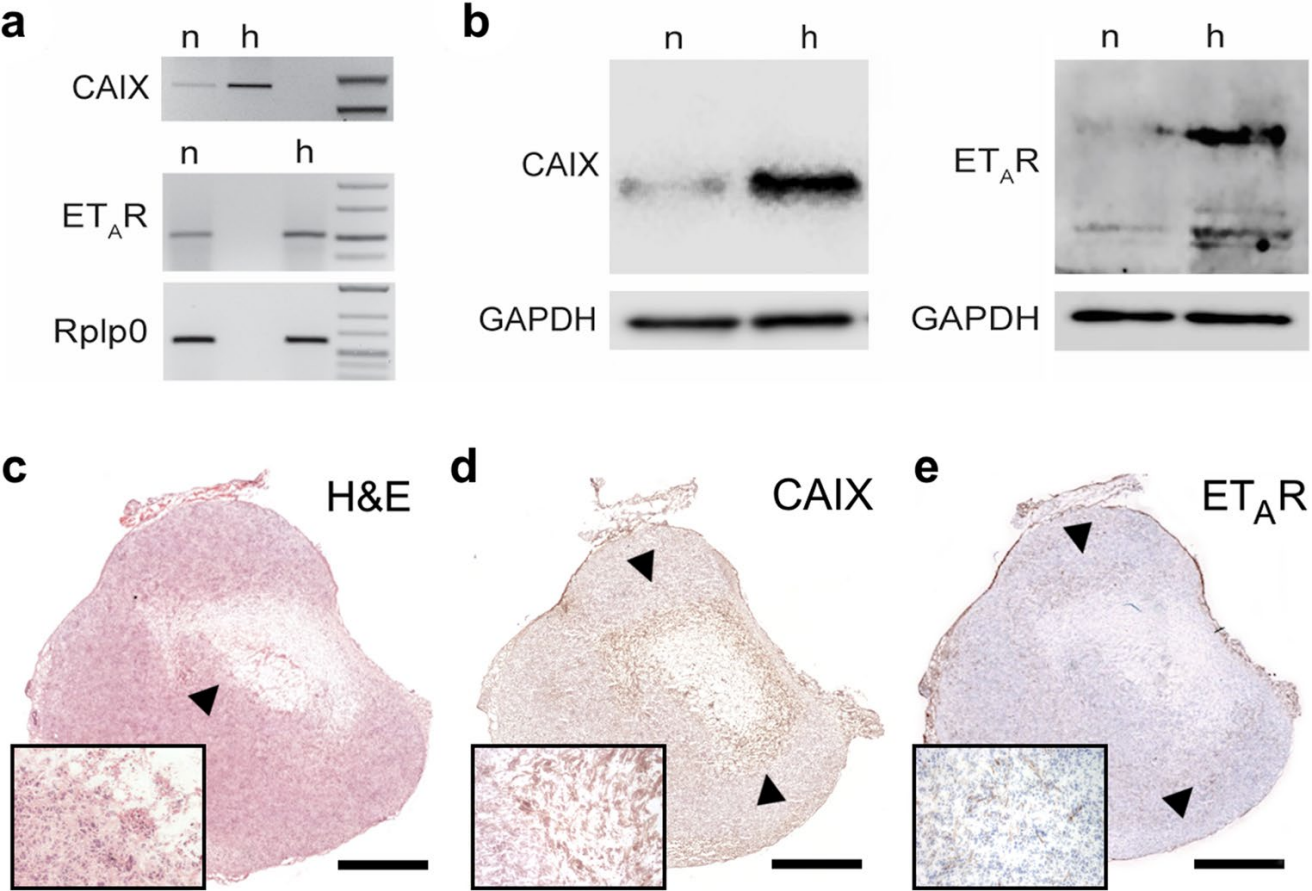
**a**-**b**. ET_A_R levels are elevated upon hypoxia. Shown are representative photomicrographs of agarose gels (**a**) and western blots (**b**) of 4T1 cells cultured under physiological (n) and hypoxic (h, 18 h 1% oxygen) conditions. CAIX mRNA (**a**) and protein (**b**) served as an indicator of hypoxia. Note the accumulation of both ET_A_R transcripts (**a**) and protein (**b**) in cells exposed to hypoxia. The 60 S ribosomal protein lateral stalk subunit P0 (Rplp0) (**a**) and GAPDH (**b**) were used as reference for RT-PCR and western blotting, respectively. **c-e**. Immunohistochemistry of a baseline tumor. **c**. Whole tumor section after H&E staining showing a central region with reduced cellularity (black arrowhead, scale bar = 1 mm). **d**. Immunostaining for CAIX as a robust marker of hypoxia, black arrowheads indicate regions of enhanced protein expression. **e**. Immunohistochemical staining for ET_A_R showing regions of high ET_A_R expression in areas adjacent to the necrotic central region (black arrowheads)

To pinpoint the relationship between ET_A_R and hypoxia, murine breast cancer 4T1 cells were cultured under hypoxic conditions (1% oxygen) for 18 h. The expression levels of membrane-bound zinc metalloenzyme carbonic anhydrase IX (CAIX), a well characterized target of HIF-1 during hypoxia [29], and ET_A_R were examined under both physiological and hypoxic culture conditions by RT-PCR and western blot analysis. Both CAIX and ET_A_R transcripts and protein levels were markedly increased in cells exposed to hypoxia (Fig. 1a, b).

### Immunohistochemical detection of ET_A_R within hypoxic tumor lesions

Excised 4T1 tumors were analyzed by immunohistochemistry, identifying CAIX and ET_A_R. Initially, the tumors showed typical characteristics of fast-growing lesions characterized by partially hypoxic, centrally located areas displaying reduced cellularity (Fig. 1c). These areas also exhibited a high expression of CAIX, in addition this protein could also be detected in areas surrounding the tumor core (Fig. 1d). Also, a number of pericentral fields showed a high expression of ET_A_R (Fig. 1e).

### MSOT-based detection and quantification of hypoxia and ET_A_R within tumor lesions

To quantitatively assess the hypoxic TME in vivo, we performed MSOT and compared baseline measurements from tumor lesions with those from healthy muscle tissue. Tumors exhibited significantly reduced signal intensities (SI) for oxygenated hemoglobin (HbO_2_, mean SI: 0.5 vs. 2.4) while those for deoxygenated hemoglobin were enhanced (Hb, mean SI: 1.1 vs. 0.4). Correspondingly, the mean oxygen saturation (sO_2_) was markedly lower within tumors compared to healthy tissue (0.34 vs. 0.79, Fig. S2a) indicative of pronounced hypoxia. Moreover, the total hemoglobin (HbT) content in these tumors was reduced by 36.7% (mean SI: 1.9 vs. 3.0).

**Fig. 2:**
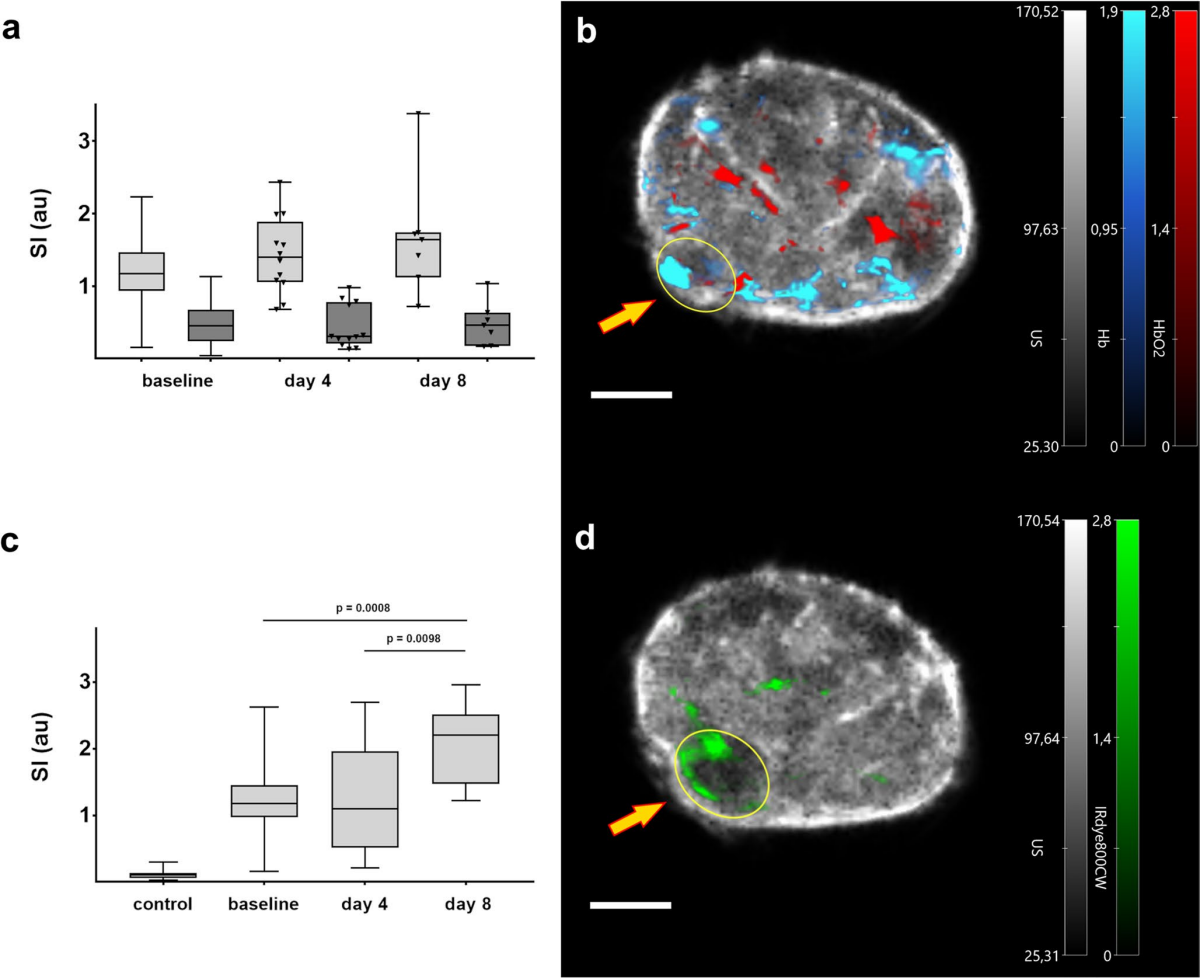
(**a**) Shown are MSOT signal intensity (SI) plots from tumors for deoxygenated (Hb, light grey) and oxygenated hemoglobin (HbO_2_, dark grey) during tumor growth compared to baseline. (**b**) Overlay of a representative pseudo color-coded MSOT image from a transversal section of the lower abdomen of the animal showing ultrasound background (grey color), oxygenated (red) and deoxygenated (blue) hemoglobin (scale bar = 5 mm). The yellow ellipse denotes a region of interest (ROI) depicting the tumor lesion (arrow). (**c**) Box-and-whisker plots of ET_A_R probe intensity compared to control (before injection of the probe) and baseline depicting the increase of ET_A_R SI during tumor growth. (**d**) Exemplary MSOT image (transversal slice, lower abdomen, scale bar = 5 mm) showing a strong signal from the ET_A_R probe in the tumor region (ellipse and arrow) at day 8 (green color, grey: ultrasound image)

To further investigate dynamic changes during tumor progression, longitudinal imaging of mice was performed over an eight-day period. MSOT enabled the delineation of Hb, HbO_2_, and the fluorescent ET_A_R probe signal intensities within tumor lesions (Fig. 2). While Hb and HbO_2_ signals showed no marked changes over time (Fig. 2a), a growth-related increase in ET_A_R probe SI was seen within tumors during the observation period (1.173 ± 0.108 ► 1.263 ± 0.227 ► 2.067 ± 0.225, Fig. 2c).

### Bevacizumab and Sorafenib therapy enhance oxygen saturation

**Fig. 3:**
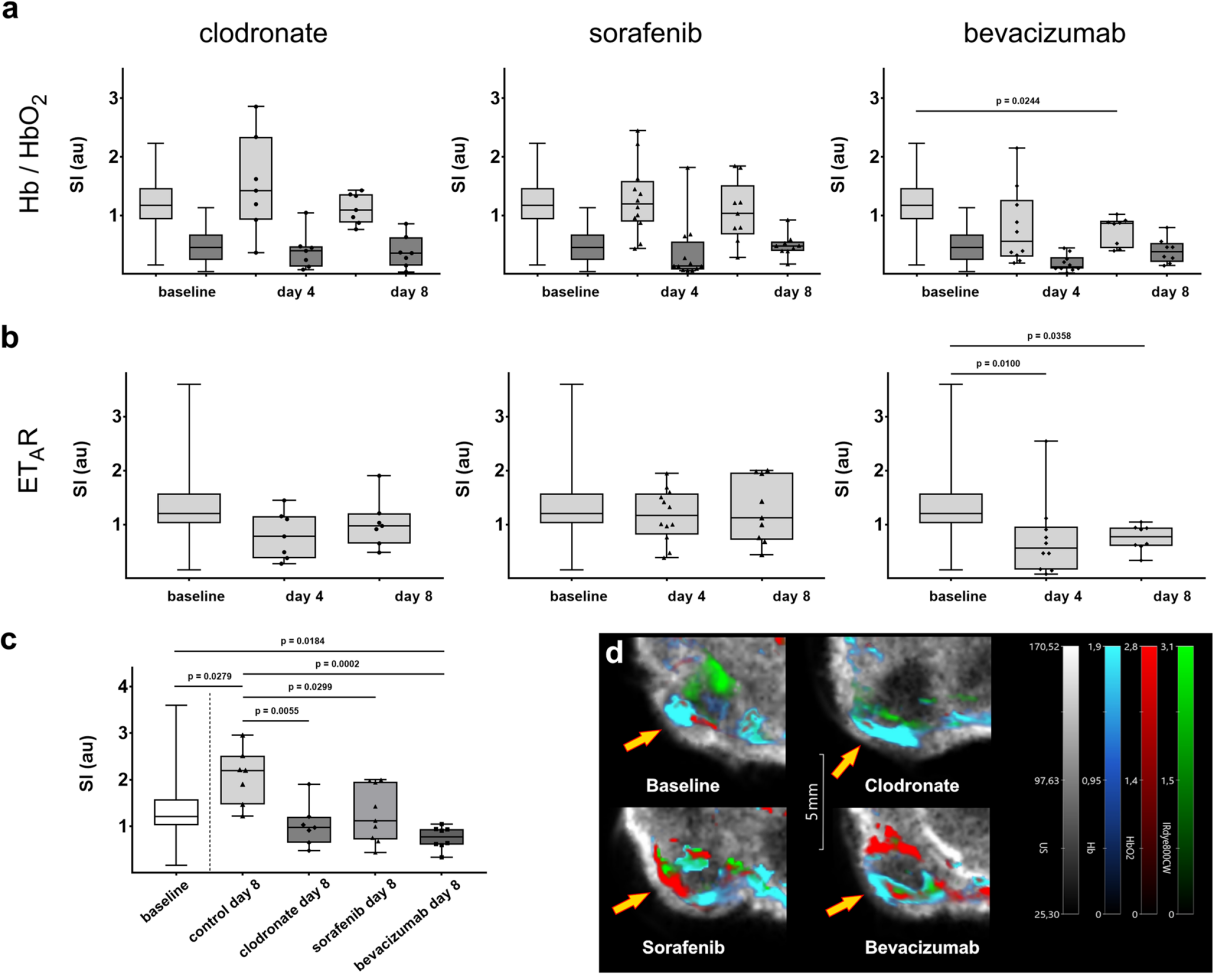
(**a**) Box plots displaying the mean MSOT SI values for deoxygenated hemoglobin (Hb, light grey) and oxygenated hemoglobin (HbO_2,_ dark grey) during therapy, compared to baseline measurements. (**b**) Box plots showing the corresponding MSOT SI values for the ET_A_R probe within tumor lesions throughout therapy (identical group order as in **a**). (**c**) Box-plots comparing MSOT SI values at the therapy endpoint to both baseline and control tumors (PBS-loaded liposomes). (**d**) Representative pseudo-colored MSOT images of the tumor region after 8 days of therapy (grey: ultrasound image, blue: Hb, red: HbO_2_, green: ET_A_R probe, arrows indicate tumor lesions)

Three different anti-cancer therapies targeting either the angiogenic potential of the tumor or its capacity to modify host immune response were applied for up to eight days (Fig. S1). *Sorafenib* is a small molecular multi-kinase inhibitor clinically approved for the treatment of primary kidney cancer, hepatocellular carcinoma and thyroid carcinoma [35]. It exerts its therapeutic effects by inhibiting the activity of VEGFR (vascular endothelial growth factor receptor), PDGFR (platelet-derived growth factor receptor) and various Raf-kinases, thereby reducing cell proliferation and tumor angiogenesis. *Bevacizumab* is a monoclonal antibody that specifically targets VEGF, regulating vascular sprouting of tumor vessels [36]. *Clodronate*, an anti-osteoporotic drug used clinically for the prevention and treatment of osteoporosis as well as pain reduction due to its potent anti-inflammatory and analgesic properties [37, 38], is also employed in preclinical studies for its macrophage-depleting capacity, thus enabling the monitoring of tumor-immune interactions [39–41]. Given that ET_A_R expression is strongly associated with the pro-tumorigenic effect of TAMs in human adenocarcinoma [30], we hypothesized that *Clodronate* might also modulate ET_A_R expression within the investigated murine breast tumor model. Therapy effects were evaluated on days 4 and 8 post-treatment initiation and compared to baseline measurements with respect to MSOT-derived values for hemoglobin and oxygen saturation (Fig. 3a). Notably, levels of deoxygenated hemoglobin exhibited substantial variability across all experiments, presumably reflecting tumor heterogeneity within the TME. Although the administered therapies did not lead to a significant reduction in tumor growth (Fig. S3), an increasing trend in HbO_2_ values from day 4 to day 8 suggests augemented oxygen supply during therapy. MSOT analysis demonstrated that treatment with *Sorafenib* (0.404 vs. 0.339, *p* = 0.0152) and *Bevacizumab* (0.402 vs. 0.339, *p* = 0.0340), both of which target VEGF signaling, resulted in a modest but statistically significant increase in sO_2_ after eight days as compared to baseline (Fig. S4b), indicating an acute pharmacological effect on oxygen supply mechanisms within the TME. For *Bevacizumab* in particular, this enhancement could be attributed to a significant reduction in deoxygenated hemoglobin (0.735 ± 0.088 vs. baseline 1.205 ± 0.062, *p* = 0.0244, Fig. 3a). In contrast, *Clodronate* therapy, which primarily affects the tumor immune landscape through macrophage depletion, did not produce marked changes in hemoglobin parameters (Fig. 3a).

### MSOT-detectable reduction in ET_A_R expression reflects treatment response

Endothelin receptors are key components of breast cancer cell signaling, being involved in cell proliferation, migration and angiogenesis [31, 32]. MSOT provides reliable data for target-associated signal intensity from the applied probe in the chosen tumor model. In control animals receiving PBS-loaded liposomes the level of ET_A_R SI significantly increased during the observation period (Fig. 2c). Application of *Clodronate*-loaded liposomes on the other hand resulted in a decrease of SI (Fig. 3b, c). After 8 days, SI of control (untreated) tumors was significantly elevated as compared to initial values, while the reduction of SI from *Clodronate*-treated tumors was significantly lower as compared to baseline already at day 4. *Sorafenib* did not show a marked effect on ET_A_R values compared to baseline, while *Bevacizumab* resulted in a rather quick decrease in ET_A_R SI already after 4 days and was still significantly lower than baseline after 8 days (Fig. 3b). When comparing the end-point of therapies after 8 days, however, all treatments showed a significant decrease of SI compared to control tumors that had grown unimpeded for 8 days (Fig. 3c). Pseudo-colored MSOT images generated using the ViewMSOT software visually illustrate the effects of the therapies. Specifically, a reduction in ET_A_R signal is evident in the *Clodronate*-treated tumors, while an increase in HbO_2_ signal is observed in tumors treated with *Sorafenib* and *Bevacizumab*, compared to baseline where strong signals from deoxygenated hemoglobin and ET_A_R were predominant (Fig. 3d).

### Therapy-induced modulation of ET_A_R, macrophage Infiltration, and vascular normalization in hypoxic tumor lesions

To assess the impact of therapy on key components of the tumor microenvironment, immunohistochemical staining was performed for ET_A_R, CAIX, CD68, and PECAM-1. These markers were selected to represent distinct biological processes implicated in tumor progression and therapeutic response. ET_A_R and CAIX were chosen to evaluate changes in endothelin signaling and hypoxic adaptation, respectively, as both are known to modulate tumor aggressiveness and resistance to therapy. CD68 was used as a marker of TAM infiltration, reflecting changes in immune cell presence within the tumor while PECAM-1 staining was used to assess vessel density and normalization in response to therapy. Collectively, this panel of markers allows a comprehensive evaluation of therapy-induced effects on signaling, hypoxia, immune cell involvement, and vascular remodeling within the TME.

**Fig. 4:**
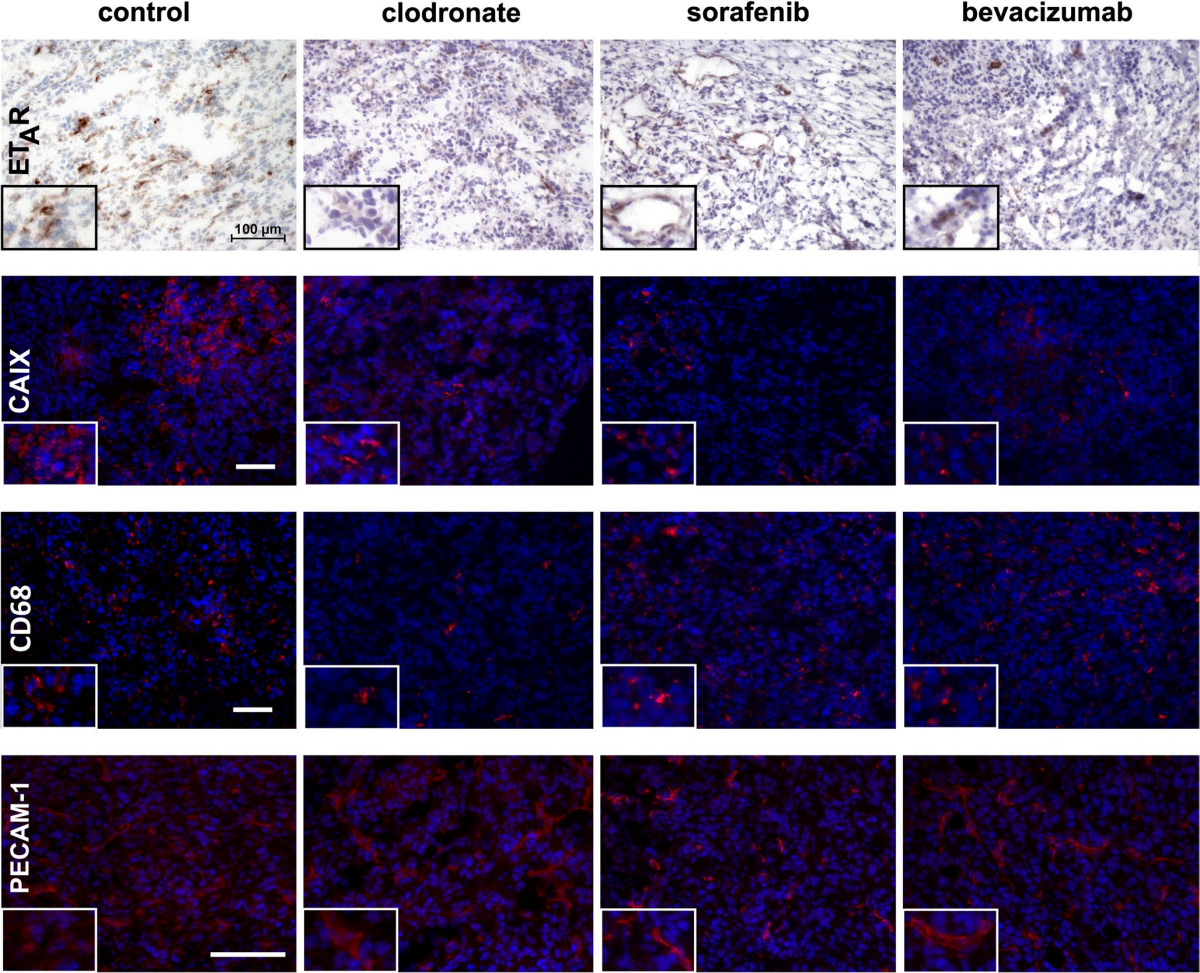
Immunohistochemistry following treatment regimens indicated at the top of each column. Representative images for CAIX and CD68 are shown at 40x magnification (scale bar = 50 μm) while those for ET_A_R and PECAM-1 are shown at 20x magnification (scale bar = 100 μm). For CAIX, CD68 and PECAM-1, nuclei were counterstained with DAPI. Insets show a 2x magnification of a relevant area

The results, shown in Fig. 4, demonstrate the differential impact of the tested therapies on expression levels of selected proteins within the TME. Tumors from animals treated with PBS-loaded liposomes served as controls and exhibited strong expression of both ET_A_R and the hypoxia marker CAIX. Following therapy, the expression of both proteins was markedly reduced. Notably, *Bevacizumab* treatment led to the most substantial decrease in ET_A_R expression, whereas *Sorafenib* most prominently diminished CAIX levels. *Clodronate* treatment also induced a pronounced reduction in ET_A_R expression, with a moderate reduction in CAIX staining that was not fully eliminated. In terms of immune cell infiltration, the reduction in CD68-positive cells, predominantly macrophages, was most prominent after *Clodronate* therapy and was less evident following *Sorafenib* or *Bevacizumab* treatment. Assessment of vascular changes revealed that vessel normalization, as assessed by PECAM-1 staining, was most clearly observed after *Clodronate* therapy (see also Fig. S5).

## Discussion

Elucidation of signaling pathways within the hypoxic TME is of crucial importance for understanding tumor biology and the development of effective therapies. Hypoxia affects a variety of signaling networks within the TME, of which the ET-axis is of particular interest. Its ability to induce stabilization of HIFs by inhibiting prolyl hydroxylases through ET receptors [33, 34], thereby supporting tumor angiogenesis, suggests a potential treatment route for solid tumors. The development of novel imaging technologies, including exogenous receptor-targeting probes, for the non-invasive in vivo monitoring of treatment response is therefore mandatory for future clinical translational approaches.

Hemoglobin in its oxygenated and deoxygenated form can be differentiated by MSOT using spectral unmixing algorithms, thus serving as an endogenous marker of tumor hypoxia and thereby finally therapy-related re-oxygenation. In a number of preclinical studies, the effect of therapies on hemoglobin levels within 4T1 tumors have been described and linked to therapy success [24, 35]. This could be directly translated to clinical studies and first proof-of-concept studies have shown feasibility [21, 36]. The high spatial resolution of MSOT allows the non-invasive in vivo detection of hypoxic tumor regions, potentially identifying particularly vulnerable areas which can be specifically targeted. Whereas clinical studies have to focus on available endogenous chromophores, preclinical in vivo examinations allow for the application of exogenous targeted probes. The used fluorescent ET_A_R probe has been demonstrated to be capable of delineating target expression changes in murine xenografts of fibrosarcoma and thyroid cancers utilizing 2D and 3D fluorescent in vivo imaging [37, 38]. Using MSOT, the ET_A_R probe showed promising responses in the visualization of target expression in human thrombendarteriectomy specimen after surgery in a proof-of-feasibility study [39]. In the present study, the effect of therapies on ET_A_R probe distribution could be visualized by MSOT and an early response in the form of a significant reduction of expression was confirmed. Especially anti-angiogenic therapies result in reduced signal intensities from the ET_A_R probe indicating vascular re-organization. This is most likely linked to downregulation of ET-1, which has been reported in glioblastoma patients [40] and patients with metastatic colorectal cancer [41] in response to *Bevacizumab* treatment. The ET-axis within the 4T1 model was investigated by Minoves et al., who showed that endothelin receptor blockade inhibits the tumor promoting effects of hypoxia in 4T1 tumors by applying the dual antagonist *Macitentan.* However, this investigation applied additional intermittent hypoxia during tumor progression [42]. In a non-cancer-associated study, Yu et al. reported that in rat mesenteric arteries, *Sorafenib* led to specific upregulation of ET_B_R, while ET_A_R was unaffected [43]. A more detailed investigation into the interrelationship between the two ET receptors within the hypoxic TME has recently become feasible with the development of a novel ET_B_R probe suitable for both fluorescence and MSOT imaging [44]. The observed reduction in ET_A_R signal following *Clodronate* therapy can be partially attributed to the expression of ET_A_R on immune cells [45, 46], which are directly depleted by this treatment. In addition, reduced de-novo synthesis of immune cells - due to altered signaling pathways– may further contribute to the observed decrease [13]. Given that elevated ET_A_R expression is associated with the presence of pro-tumoral TAMs [30], these findings underscore the therapeutic potential of targeting the ET-axis within the TME.

Some limitations of our study should be considered. First, we observed a rather brief period of accelerated tumor growth, likely resulting in an accentuated hypoxic TME; such conditions may not accurately reflect the heterogeneity and progression observed in real-life cancer biology. This limitation is further compounded by the inherent variability within the collected MSOT data, particularly in measurements of hemoglobin content within tumor lesions. The optoacoustic signal is highly sensitive to local tissue properties - such as optical scattering, tissue heterogeneity within the TME, and differences in probe positioning and animal handling - which can introduce significant data variability and pose challenges to reproducibility and interpretation. A reliance on solely endogenous contrast limits the specificity of hypoxia evaluation. In addition, a standardization of data acquisition protocols and analysis algorithms for assessing hypoxia by optoacoustic imaging is currently lacking in the field, making cross-study comparisons difficult.

Expanding the sample size in future studies could help to address data heterogeneity and improve statistical robustness. Furthermore, integrating an additional therapeutic regimen targeting the ET-axis would provide valuable mechanistic insights into the pathways implicated. A more in-depth analysis of these molecular pathways, ideally supported by innovative imaging modalities such as MSOT, may deepen our understanding of tumor biology and pave the way for novel therapeutic alternatives in the future. Recent advances, such as fluorescence-guided surgery using dye-labeled targeted antibodies for intraoperative tumor margin detection [47], highlight the translational potential for exogenous contrast agents. Their integration with non-invasive optoacoustic imaging could enhance the clinical applicability of MSOT. Continued technological and methodological refinement - including calibration, fluence correction, and standardization - will be essential to ensure the reproducibility, sensitivity, and overall utility of MSOT in both research and clinical contexts.

## Conclusions

Here, we show, that an exogenous ET_A_R targeting fluoroprobe is more precise than using the endogenous fluorophore hemoglobin in detecting tumor growth and hypoxia using MSOT in a model of murine breast cancer. Even at early stages during tumor progression, when no response to therapy in terms of macroscopic tumor growth can be detected using conventional imaging methods like CT or MRI, our study reveals significant changes in TME composition, which in part also indicate a re-oxygenation of those areas, enabling mapping of these changes in vivo. Furthermore, our results demonstrating an elevated expression of ET_A_R under hypoxic stress and a reduction following treatment suggest that ET_A_R might be used as an early prognostic marker for tumor progression.

## Supplementary Information

Below is the link to the electronic supplementary material.

Supplementary Material 1 (DOCX 684 KB)

Supplementary Material 2 (PNG 779 KB)

High Resolution Image (TIFF 219 KB)

## Data Availability

Data and images (raw data) generated during this current study are available from the corresponding author on reasonable request.
