# Supplementary material for: In vivo optoacoustic imaging of endothelin receptor expression and treatment response in the hypoxic tumor microenvironment
Source: Eur J Nucl Med Mol Imaging. 2025 Aug 13;53(2):1331–42. doi: 10.1007/s00259-025-07494-7 (PMC12830490; doi:10.1007/s00259-025-07494-7)
Supplement: Supplementary file 1 — Supplementary Material 1 (DOCX 684 KB) [file 259_2025_7494_MOESM1_ESM.docx]

***In vivo* Optoacoustic Imaging of Endothelin Receptor Expression and Treatment Response in the Hypoxic Tumor Microenvironment**

Carsten Höltke^1*^, Moushami Mallik^1*^, Miriam Stölting^1^, Emily Hoffmann^1^, Christiane Geyer^1^, Raghu Erapaneedi^2^, Friedemann Kiefer^2^, Anne Helfen^1^

^1^ Clinic for Radiology, University of Muenster and University Hospital Muenster, Muenster, Germany

^2^ European Institute for Molecular Imaging (EIMI), Multiscale Imaging Center (MIC), University of Muenster, Muenster, Germany

^*^ These authors contributed equally.

**Corresponding author:** Anne Helfen, Clinic for Radiology, University Hospital Muenster, Albert-Schweitzer-Campus 1, A1, D-48149 Muenster, T +49 251 83 44199, F +49 251 83 49656, (anne.helfen@ukmuenster.de), reprints should be directed to Anne Helfen.

**First authors:** Carsten Höltke & Moushami Mallik, Clinic for Radiology, Multiscale Imaging Center, Röntgenstraße 16, D-48149 Muenster, T +49 251 83 39673, F +49 251 83 49656.

**Supplementary Information File**

**Contents:**

1. Supplementary Figures

Figure S1: Schematic illustration of the study design and therapeutic regimen (partially created with BioRender.com)

Figure S2: MSOT-Based Evaluation of Hemoglobin Species, Oxygen Saturation, and Data Processing Approaches

Figure S3: Therapy-induced effects on tumor size dynamics

Figure S4: Effects of therapeutic interventions on MSOT-derived Hemoglobin and sO₂

Figure S5: Assessment of PECAM-1 expression in tumor tissue following eight days of therapy

1. Supplementary Methods: Real-Time PCR, Western Blotting and PECAM-1 quantification.
2. **Supplementary Figures**


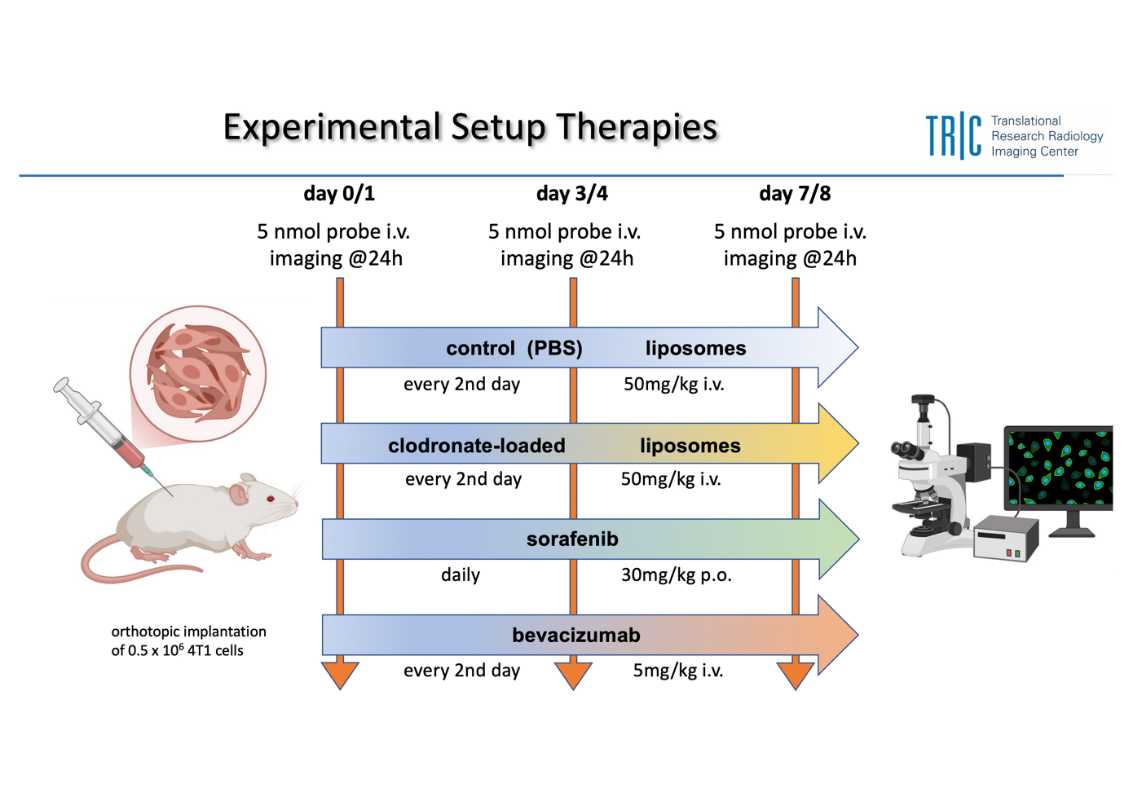


**Fig. S1** Schematic illustration of the study design and therapeutic regimen (partially created with BioRender.com)


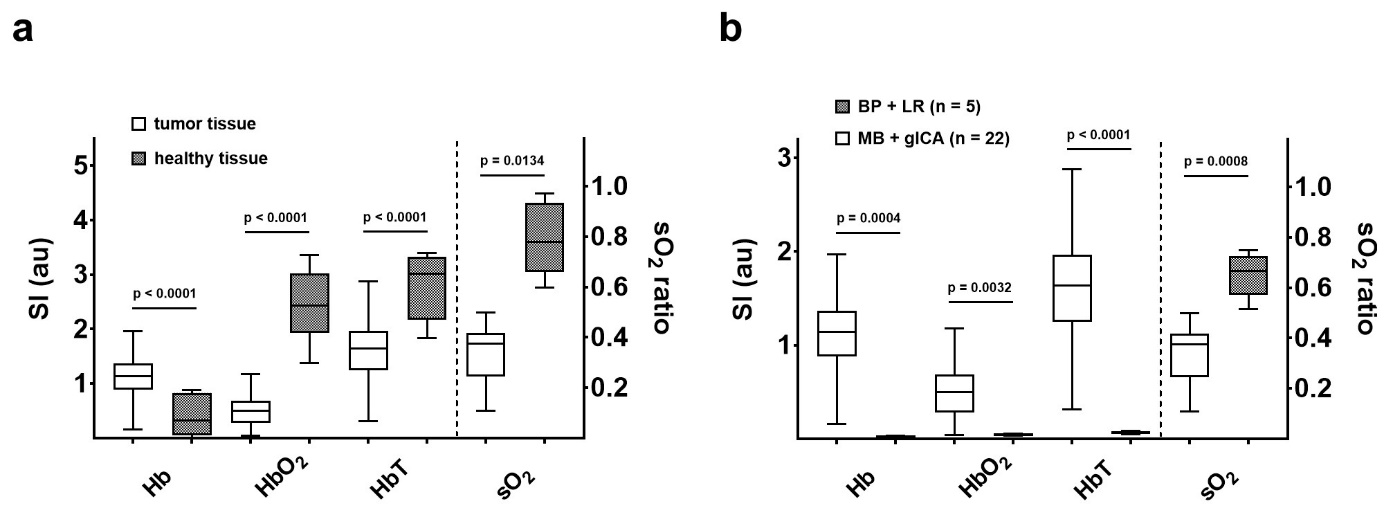


**Fig. S2** MSOT-Based Evaluation of Hemoglobin Species, Oxygen Saturation, and Data Processing Approaches

MSOT SI (left y-axis) plots for deoxygenated (Hb), oxygenated (HbO2), and total hemoglobin (HbT) in tumors (n = 12, white boxes) and healthy muscle tissue (n = 8, patterned boxes). Mean oxygen saturation (sO2) values for tumors and healthy muscle tissue were calculated and are shown on the right y-axis (a). SI (left axis) for Hb, HbO2, and HbT in tumor tissue, determined using backprojection and linear regression algorithms (BP + LR, patterned boxes) is compared to values obtained from model-based reconstruction and guided ICA analysis (white boxes, same values as in a) (b). Corresponding sO2 values from both methods are also shown on the right y-axis (b). Notably, hypoxic status of the tumor cannot be determined from values obtained from backprojection and linear regression analysis alone.

**Fig. S3** Therapy-induced effects on tumor size dynamics

Longitudinal comparison of tumor size throughout the imaging experiments under different therapy conditions. Tumor volumes were measured at defined timepoints to monitor growth and treatment response.

**
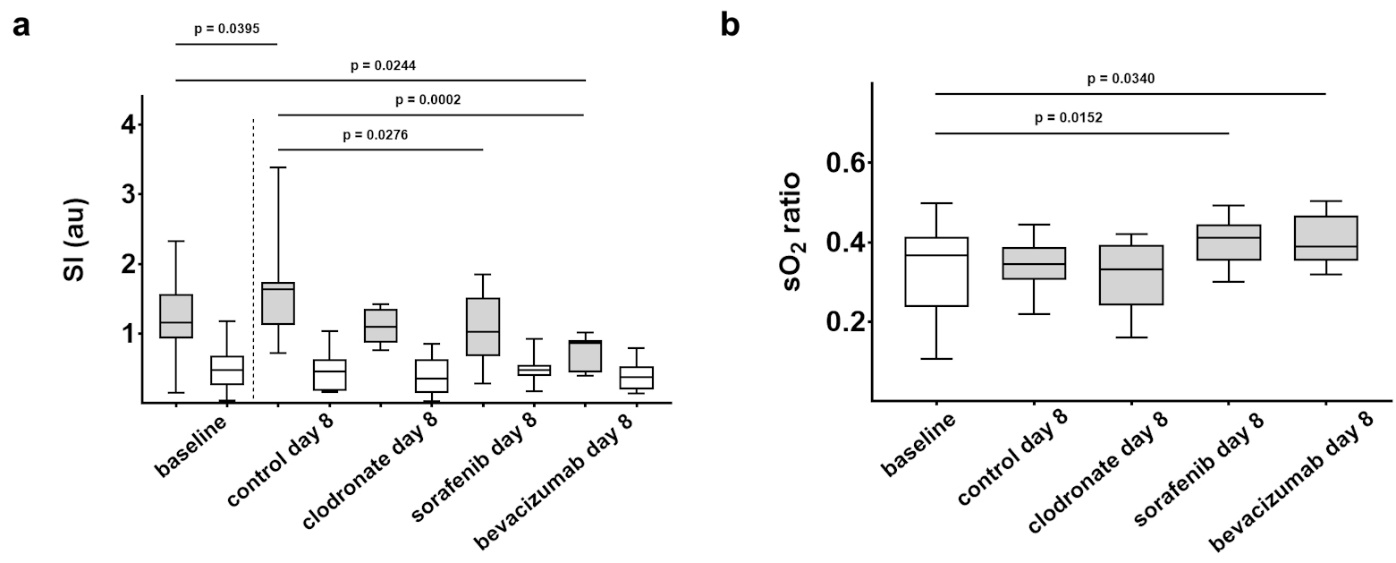
**

**Fig. S4** Effects of therapeutic interventions on MSOT-derived Hemoglobin and sO₂

Quantification of MSOT-derived values for deoxygenated hemoglobin (Hb) and oxygenated hemoglobin (HbO₂) (**a**), and the corresponding calculated sO₂ ratios (**b**) at therapy endpoints, compared to baseline and control groups.

**Fig. S5** Assessment of PECAM-1 expression in tumor tissue following eight days of therapy

1. **Supplementary Methods**

**Real-Time PCR**

4T1 cells (1 x 10^6^) were exposed to acute hypoxia (1 % O_2_) for 18 h. Following treatment, normoxic and hypoxic cells were centrifuged at 300 g at 4°C for 7 min and total RNA was isolated using the RNeasy mini kit with on-column DNase I digestion according to the manufacturers’ instructions (Qiagen, Hilden, Germany). Reverse transcription was performed using 2 µg of total RNA with the Quantitect reverse transcription kit as described by the manufacturer (Qiagen). ET_A_R (QT00121625, Qiagen), CAIX (For: 5’-acggggatgaaaaagggggt-3’ and Rev: 5’-gtaccgtgtggccattgttg-3’) and Rplp0 (QT00249375, Qiagen) were used for amplification of cDNA using 2x DreamTaq PCR master mix in an Eppendorf thermocycler. RT-PCR products were electrophoresed on a 2% agarose gel and visualized using a GelJet imager (INTAS Science Imaging, Göttingen, Germany).

**Western Blotting**

4T1 cells (0.5 x 10^6^) were exposed to acute hypoxia (1 % O_2_) for 18 h. Following treatment, normoxic and hypoxic cells were centrifuged at 300 g at 4 ⁰C for 7 min and the cell pellet was lysed in buffer containing 150 mM NaCl, 50 mM Tris, 1% Triton X-100, 1 mM EDTA, 1 mM EGTA, 30% Glycerin and 10 mM Na_3_VO_4_ at 4 ⁰C overnight. Equal amount of proteins was loaded in each lane after determining total protein concentrations using Bradford reagent (AppliChem, Darmstadt, Germany) and subjected to electrophoresis using 12 % SDS-PAGE and transferred to PVDF membrane (Immobilon-P transfer membranes, Millipore). Molecular weight markers (Pageruler plus prestained protein ladder, Thermo Fisher Scientific) was loaded in a separate lane. The membrane was then incubated for 30 mins in blocking buffer for fluorescent Western blotting (Rockland Immunochemicals, Pottstown, PA, USA) at RT. Next, the membrane was incubated overnight at 4 ⁰C with primary antibodies, followed by incubation with IRDye800CW goat anti-rabbit IgG (1:20,000) secondary antibody for 1 h at RT. After each incubation, the membrane was thoroughly washed with TBST (3 x 10 mins). Protein signals were visualized using the LI-COR Odyssey XF imager.

**PECAM-1 staining quantification**

For quantification, images of PECAM-1 stained tumor slides (n = 5-10 for each group) were converted to binary images with Adobe Photoshop, re-sized and digitally placed under a transparent grid containing a crosshair. This crosshair was used to divide the image into four equal quadrants. The grid has boxes/squares sized 8 × 8 pixels = 100 μm^2^. For quantification, a sector of 200 μm on the Y-axis and 280 μm on the X-axis was defined equaling 56,000 μm^2^ divided into 560 boxes. Presence versus absence of a piece of vessel in the defined boxes was scored and calculated as a measure of relative vessel coverage. All measurements were performed by a single observer masked to the slide identity.
