# Supplementary figures and images for: In vivo optoacoustic imaging of endothelin receptor expression and treatment response in the hypoxic tumor microenvironment
Source: Eur J Nucl Med Mol Imaging. 2025 Aug 13;53(2):1331–42. doi: 10.1007/s00259-025-07494-7 (PMC12830490; doi:10.1007/s00259-025-07494-7)

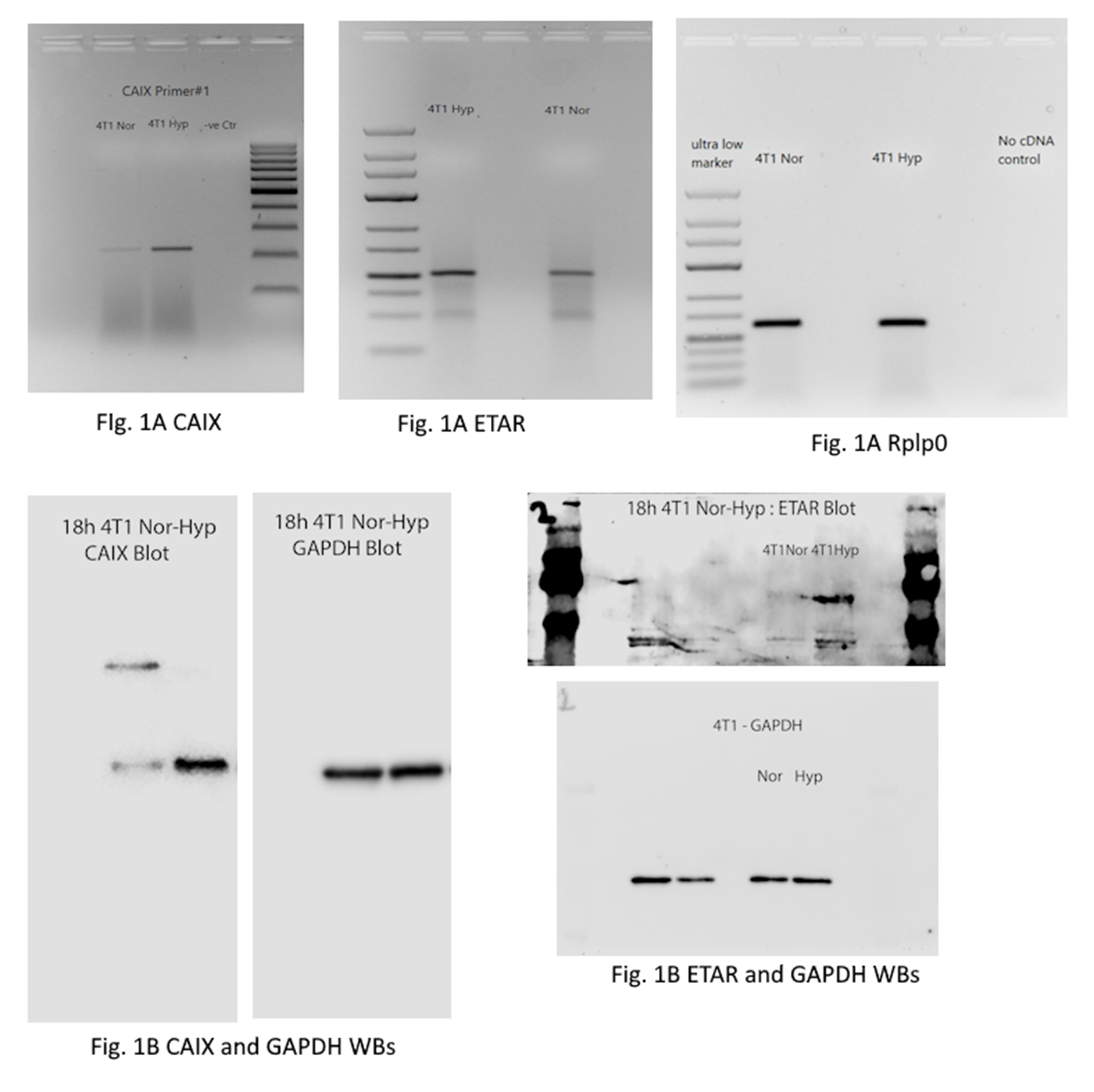

Supplement: Supplementary file 2 — (PNG 779 KB) [file 259_2025_7494_Fig5_ESM.png]

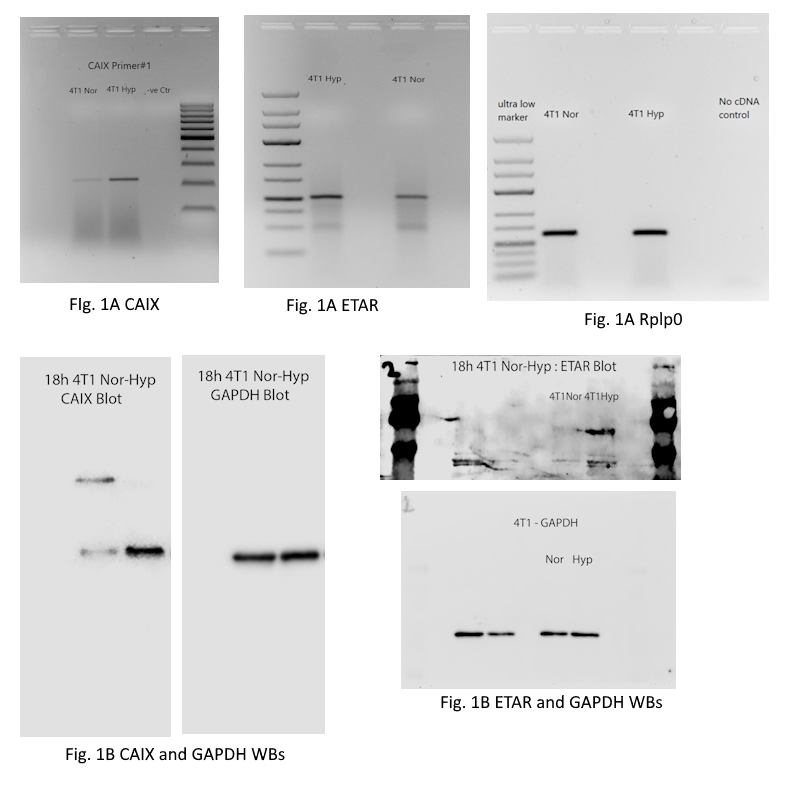

Supplement: Supplementary file 3 — High Resolution Image (TIFF 219 KB) [file 259_2025_7494_MOESM2_ESM.tiff]
